# Long-term survival and quality of life in Jehovah’s witnesses after cardiac surgery: a case control study

**DOI:** 10.1186/s12872-019-1061-z

**Published:** 2019-03-29

**Authors:** Pierre Wauthy, Charalampos Pierrakos, Louis Chebli, Redente Tortora

**Affiliations:** 1 Department of Cardiac Surgery, CHU Brugmann, Place A van Gehuchten, 1020 Brussels, Belgium; 2 Intensive Care Unit, CHU Brugmann, Université Libre de Bruxelles, Brussels, Belgium

**Keywords:** Bleeding, Blood transfusion, Cardiopulmonary bypass, Quality of life

## Abstract

**Background:**

We previously analyzed morbidity and mortality in Jehovah’s Witnesses patients after cardiac surgery compared to control population patients. Patients who were Jehovah’s Witnesses were operated in accordance with their philosophical convictions and in respect of their refusal of transfusions. We propose to assess long-term survival and quality of life in the patients of this preliminary study.

**Methods:**

We contacted 31 adult Jehovah’s Witnesses patients who underwent heart surgery at the Brugmann hospital between 1991 and 2012 and compared them to a control population of 62 patients that had no transfusion restriction, and matched them for sex, age at the time of intervention and the type of surgery performed. We compared long-term quality of life in both populations through the MacNew software, a validated instrument to assess quality of life of patients with cardiovascular disease. The long-term survival of patients was analyzed by Kaplan Meier curves.

**Results:**

Long-term quality of life and survival do not appear different between the two groups. Patient evaluation by MacNew software shows comparable physical (*p* = 0.54), emotional (*p* = 0.12), social (*p* = 0.21) and global (*p* = 0.25) scores between the two populations. The analysis of the actuarial survival curves shows no differences in terms of long-term survival of these patients (*p* = 0.37).

**Conclusions:**

Cardiac surgery in Jehovah’s Witnesses can be performed with identical long-term quality of life and survival compared to surgery without blood transfusion restriction, if one follows rigorous blood conserving strategies.

**Trial registration:**

NCT03348072. Retrospectively registered 16 November 2017.

## Background

Cardiac surgery in Jehovah’s Witnesses (JhW) represents a real challenge for surgeons, anesthesiologists and other medical teams. The use of advanced blood saving techniques makes it possible to propose to these patients cardiac procedures with a risk of morbidity and mortality revealed in case-control studies with almost the same number [1–4].

Nowadays, the use of a health-related quality of life questionnaire is very widely recommended to evaluate a medical practice [5]. The evaluation of the long-term health-related quality of life of patients who have received treatment has become the golden standard in evaluating the effectiveness of these treatments [5]. Patient quality of life assessment tools should be validated for the pathology investigated, in the language used to administer these questionnaires. The evaluation of the quality of life of patients who have undergone heart surgery can be performed using the MacNeW questionnaire [5] which has been validated in French [6].

The determinants of quality of life after cardiac surgery are poorly studied and poorly understood. Work analyzing these parameters in patients with cardiac surgery has evolved recently. It has been shown that the quality of life of patients after cardiac surgery has improved, including for older patients [7]. One of the pre-operative determinants of this improvement is the preoperative quality of life of the patients [8]. Recently, it has been reported that intensive care unit length of stay can significantly influence the quality of life of patients [9, 10].

We propose to evaluate long term survival and health-related quality of life of two cohorts of patients operated on the heart, one without transfusion restriction, the other refusing transfusions (Jehovah’s Witnesses patients). These two cohorts of patients have already been the subject of a study evaluating early postoperative morbidity and mortality [Marinakis 2016].

## Methods

The study was conducted in the Department of Cardiac Surgery at the University Hospital Brugmann in Brussels. The Hospital’s Ethics Committee reviewed the study protocol and gave its agreement for the achievement of the study (CE 2016/23). The process of identifying JhW patients and controls has previously been detailed, as well as details of the anesthesiology and surgical management [2]. The control patients were matched for sex, age at time of surgery and the type of surgery performed. The control group was selected based on the type of surgical procedure to keep this variable constant, which can influence the comparison of outcomes.

The analysis of the quality of life in patients investigated was made with the MacNew: a heart disease Health-Related Quality of Life (HRQL) questionnaire. After patients gave their informed consent to study participation, the questionnaires were sent by post and self-administered by the patients. The results were collected over a telephone interview.

The evaluation is done by means of a questionnaire based on daily life during the 2 weeks prior and consists of 27 issues investigating the physical, emotional and social spheres of everyday life. Each domain is rated on a 7 points scale: 1 indicates poor HRQL and 7 excellent HRQL. Finally a global score, rated on a 7 points scale obtained by means of an average of each score, is determined. Mortality data and the quality of life questionnaire were collected by telephone interview. In the absence of response, the patient’s physician was contacted to confirm his survival or death.

We performed an analysis of data using the Statistical Package for Social Science software v. 22 (SPSS Inc., Chicago, IL, USA) to compare JhW to control groups. Kolmogorov-Smirnov test was applied to verify the normality of the distribution of the continuous variables. Continuous variables were compared using Student t test or Mann Whitney test as appropriate. Dichotomous variables were compared using the χ^2^ test. Survival was analyzed using the Kaplan Meier method and the data were compared with the log-rank test. Median follow up time was estimated as the time corresponding to the 50th percentile taken from reverse Kaplan Meier analysis. Statistical significance was defined as *p* < 0.05.

## Results

JhW group consists of 31 patients who underwent a variety of cardiac surgeries including elective and emergency surgery. The demographic characteristics of the patients are shown in Table 1. Surgical procedures were coronary artery bypass grafting in 55%, valvular surgery in 29%, combined coronary and valvular surgery in 10% and others in 6%. We compared them to a control group of 62 patients. The demographic characteristics of Jehovah’s Witnesses versus the controls are presented in Table 1. Patients were operated between January 1991 and December 2012. The minimum time from operation till questionnaire administration in both groups was 4 years (December 2016).

**Table 1:** Patients demographics

	Jehovah’s Witnesses (*n* = 31)	Control Group (*n* = 62)	*p* Value
Age (years)	62 ± 15	62 ± 14	0.873
Women BMI (kg/m^2^)	10 (32%) 27.4 ± 4.3	20 (32%) 26.2 ± 4.0	1.000 0.182
Euroscore II	2.80 ± 3.34	2.38 ± 2.20	0.469
Preoperative Cockcroft’s CC in mL/min	84 ± 29	82 ± 27	0.776
Renal insufficiency (Cockcroft’s CC < 85 mL/min)	16 (52%)	34 (55%)	0.769
Diabetes	8 (26%)	15 (24%)	0.865
Recent smoker	3 (10%)	17 (27%)	0.062
Hypertension	18 (58%)	30 (48%)	0.379
Hypercholesterolemia	19 (61%)	36 (58%)	0.765
Positive family history	10 (32%)	18 (29%)	0.749
Cerebrovascular accident	4 (13%)	5 (9%)	0.457
Peripheral vascular disease	8 (26%)	6 (10%)	0.040
Previous myocardial infarction	6 (19%)	11 (18%)	0.850
Recent myocardial infarction (< 90 days)	3 (10%)	6 (10%)	1.000
COPD	6 (19%)	6 (10%)	0.189
NYHA			0.452
I	6 (19%)	7 (11%)	–
II	12 (39%)	21 (34%)	–
III	10 (32%)	30 (48%)	–
IV	3 (10%)	4 (6%)	–
Preoperative LVEF	62 ± 11	59 ± 14	0.340
Preoperative AF	5 (16%)	9 (15%)	0.838
Reoperation	3 (10%)	8 (13%)	0.650
Urgent status	7 (23%)	6 (10%)	0.091

(*BMI* Body Mass Index, *CC* Creatinine Clearance, *Recent smoker* Current smoker or ex-smoker for less than 5 years, *COPD* Chronic Obstructive Pulmonary Disease, *NYHA* New York Heart Association score, *AF* Atrial Fibrillation, *LVEF* Left Ventricular Ejection Fraction)

**Fig. 1:**
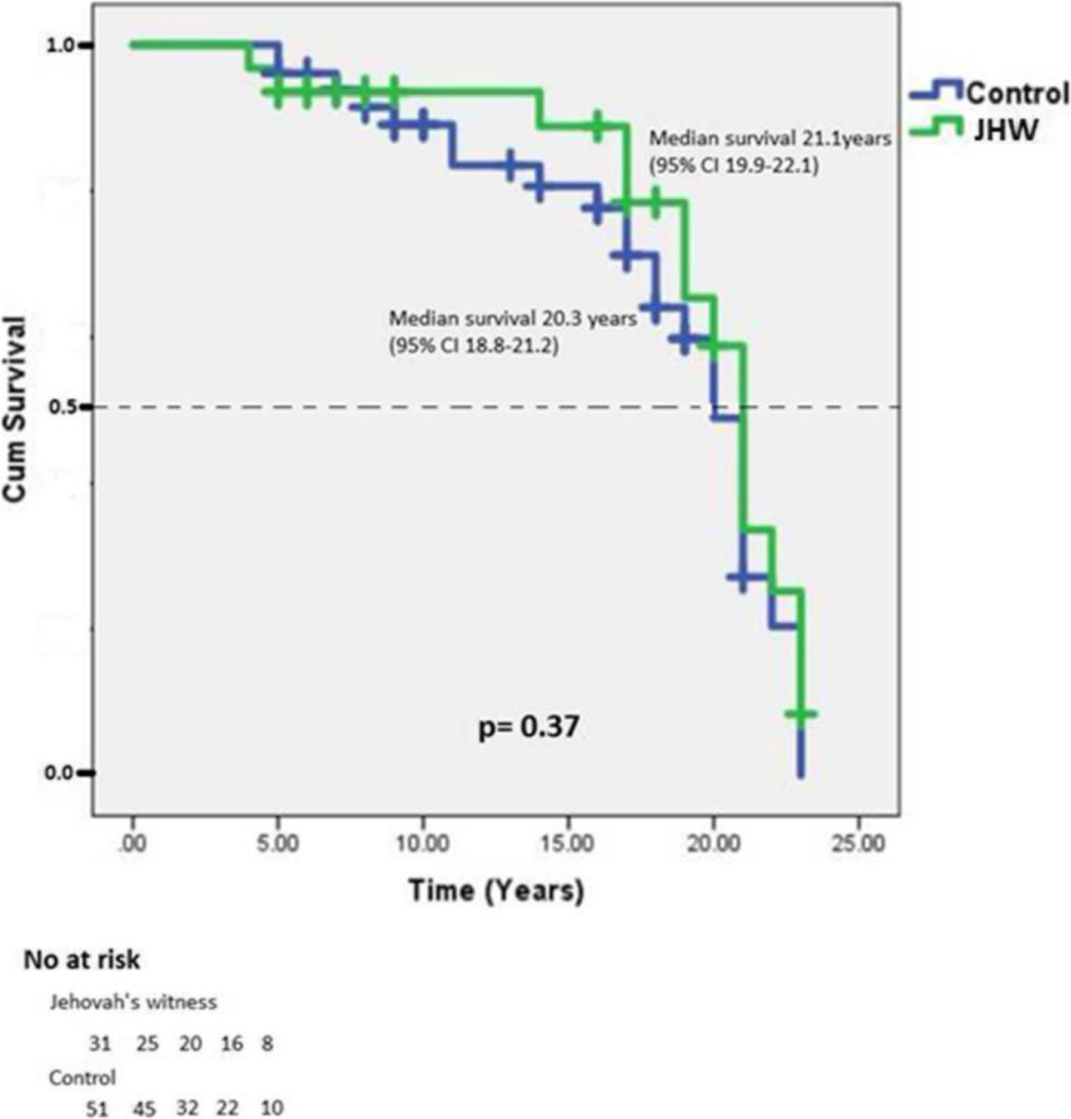
Kaplan Meier curves in the JhW (green) group and the control group (blue): *p* = 0.37

Among the 93 patients included in this study, 11 patients were lost to follow-up (all in the control group). Of the 82 remaining patients, 38 patients had died (14 in the JhW group and 24 in the control group (*p* = 0.99)) and 44 survived. The median follow-up were 15.3 years (95%CI 12–17) versus 15.9 years (95%CI 14–17) in the JhW vs control group respectively. The actuarial survival curves of the two groups are completely superimposed: no significant difference in survival was demonstrated by analysis of Kaplan Meier curves (Fig. 1). Median estimated survival and survival at 0/5/10/15 and 20 years are expressed in Fig. 1. There was no difference between the two groups (*p* = 0.37).

**Fig. 2:**
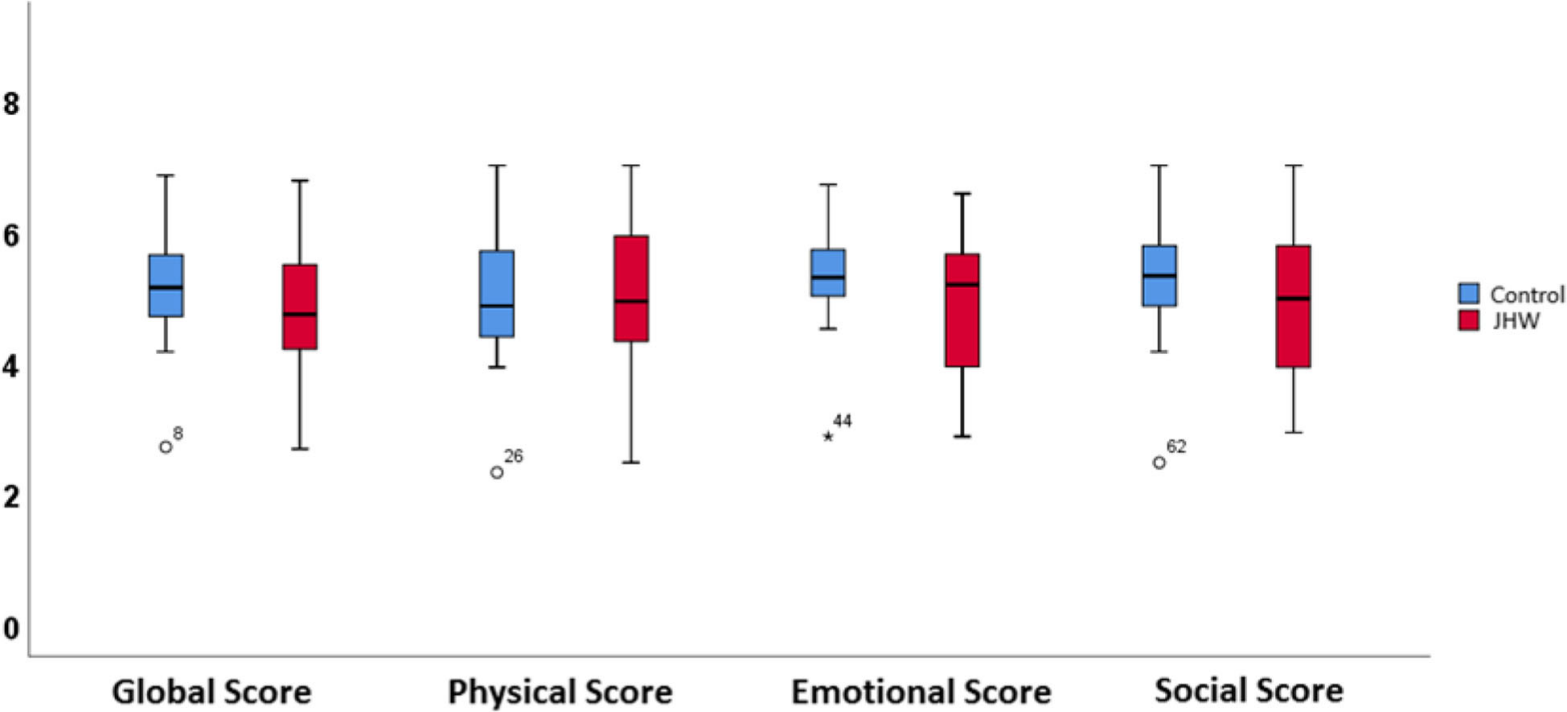
Global, Physical, Emotional aud Social scores in the MacNEW evaluation scale

The evaluation of quality of life was based on the 44 survivors (17 JhW and 27 controls). Three refused to answer the MacNew quiz (JhW 2 and 1 control) and 9 others were unable to respond due to dementia (JhW 1 and 8 control). Therefore, 32 patients in total participated in the evaluation of the quality of life with the MacNew (JhW 14 patients and 18 control). We compared between groups the delay between surgery and evaluation (*p* = 0.23) and age at the time of evaluation (*p* = 0.15). The MacNew scores were similar for both populations. Results are expressed in Fig. 2. The results are expressed in absolute value in Table 2.

**Table 2:** MacNew® score

	JhW Group (*n* = 14)	Control Group (*n* = 18)	*p* Value
Physical score	4.9 ± 1.2	5.2 ± 1.1	0.54
Emotional score	4.7 ± 1.1	5.3 ± 0.9	0.12
Social score	4.9 ± 1.1	5.4 ± 1.1	0.21
Global score	4.8 ± 1.1	5.2 ± 1.1	0.25

JhW: Jehovah’s Witness

## Discussion

The assessment of HRQL is an increasingly important outcome in the management and care of cardiac patients [5]. Quality of life is a very broad concept, multidimensional and subjective which can be difficult to evaluate correctly. The MacNew questionnaire is a specific instrument for cardiovascular disease [5, 11]. It takes into account physical, psychological, relational as well as more specific issues such as sexuality and self-image. [5, 11]. Currently validated in 23 languages, the MacNew is a reference tool in many studies. It intervenes in setting the therapeutic point of coronary heart disease (medical treatment, percutaneous angioplasty, bypass surgery, implantable defibrillator, pacemaker) in rehabilitation strategies and secondary prevention after heart failure, myocardial infarction, angina and cardiac surgery. Our evaluation method had to be adapted to French speaking patients, which is the case of MacNew [6]. This questionnaire was also validated after rehabilitation in cardiac surgery [12]. In the literature there are several discussions regarding the modality of administration of this type of questionnaire (interview, self-administration). Some studies conclude that different modes of administration of questionnaire do not lead to the same understanding of the issue and may affect the response provided. Therefore, care must be taken in interpreting the results in the administration of the questionnaire. We have been attentive to this aspect and have taken the necessary precautions so that the mode of administration of the questionnaire is identical for each patient of our study.

Many studies show the feasibility of cardiac surgery in JhW patients [13–16] but very few evaluate its long-term results. The only long-term outcome evaluated in a cohort of 322 patients over a 28-year period was based on patient survival and the occurrence of new cardiac events [3]. For the first time we report an evaluation of the quality of life of JhW patients after heart surgery and compare it to a control population. We lack the data for the 11 patients lost to follow-up. We could not assess the quality of life in 9 patients with dementia too advanced for them to respond to the interview. However, we believe that this condition has no connection with the refusal of transfusion during surgery: only one of them was a JhW. The quality of life assessed by the questionnaire was identical in the JhW population and the control population. The scores observed in both groups are close to the scores reported in the literature in patients after rehabilitation for treated coronary events [6].

There are many methods to analyze the long-term mortality of a population. The method used here is considered as the most robust: a reversed Kaplan Meier survival curve. The obtained prediction curve extends to a post-operative period of about 25 years. In the current data, we have a postoperative follow-up period ≥20 years in only 4 patients and between 10 and 20 years in 33 patients. The 45 remaining patients have a follow-up ≤10 years. The actuarial survival curves of JhW patients and control patients are totally superimposable. The survival observed after 20 years is less than reported in the literature: 10% vs 32% [3]. This can be explained by the fact that JhW patients we studied differs significantly from other studies for surgeries performed. In our study, we find a wide variety of surgical procedures at high risk, including emergencies, reoperations and combinations of several techniques at the same operation. Anyway, our JhW population has the same life expectancy as our control population.

### Limitation of our study

One must consider in the interpretation of the results that both groups are small and heterogeneous. This is particularly relevant for the assessment of the quality of life: only 14 and 18 patients responded to the MacNew questionnaire in the JhW and control group respectively. Future multi-institutional series are highly recommended to support our results and conclusions.

## Conclusions

Overall long-term quality of life and survival in the JhW group were similar to the control group.

## Abbreviations

HRQL: Health-related quality of life
JhW: Jehovah’s witnesses
SPSS: Statistical package for the social sciences
